# Rising CO_2_, Climate Change, and Public Health: Exploring the Links to Plant Biology

**DOI:** 10.1289/ehp.11501

**Published:** 2008-09-19

**Authors:** Lewis H. Ziska, Paul R. Epstein, William H. Schlesinger

**Affiliations:** 1 U.S. Department of Agriculture Agricultural Research Service, Crop Systems and Global Change Laboratory, Beltsville, Maryland, USA; 2 Center for Health and the Global Environment, Harvard Medical School, Boston, Massachusetts, USA; 3 Cary Institute of Ecosystems Studies, Millbrook, New York, USA

**Keywords:** aerobiology, contact dermatitis, food security, pharmacology, toxicology

## Abstract

**Background:**

Although the issue of anthropogenic climate forcing and public health is widely recognized, one fundamental aspect has remained underappreciated: the impact of climatic change on plant biology and the well-being of human systems.

**Objectives:**

We aimed to critically evaluate the extant and probable links between plant function and human health, drawing on the pertinent literature.

**Discussion:**

Here we provide a number of critical examples that range over various health concerns related to plant biology and climate change, including aerobiology, contact dermatitis, pharmacology, toxicology, and pesticide use.

**Conclusions:**

There are a number of clear links among climate change, plant biology, and public health that remain underappreciated by both plant scientists and health care providers. We demonstrate the importance of such links in our understanding of climate change impacts and provide a list of key questions that will help to integrate plant biology into the current paradigm regarding climate change and human health.

The concentration of atmospheric carbon dioxide has increased by 22% since 1960 to a current background level of approximately 385 μmol/mol (Intergovernmental Panel on Climate Change 2007). Recent evidence that the growth rate of CO_2_ emissions may have jumped from 1.3% to 3.3% per year from the 1990s to 2000–2006, potentially as a result of declining global sinks and increased economic activity, emphasizes the critical need to characterize the probable impacts of this impending climate forcing on human systems (Canadell et al. 2007).

Because CO_2_ absorbs heat leaving the earth’s atmosphere, there is widespread agreement that increasing CO_2_ is projected to result in increasing surface temperatures and wider swings in weather. The extent to which temperatures increase and weather patterns shift and the potential consequences for human health, from heat-related deaths to the spread of vector-borne diseases, have been addressed in the scientific literature (Epstein 2005; Gamble et al. 2008; Patz and Kovats 2002). Here we describe additional dimensions of global environmental change: the response of terrestrial plants to the buildup of atmospheric CO_2_, potential climatic forcing with respect to temperature on plant growth, and the implications for human health and nutrition.

Plant biology is directly affected by rising CO_2_ because CO_2_ is the sole supplier of carbon for photosynthesis. Because approximately 95% of all plant species are deficient in the amount of CO_2_ needed to operate at maximum efficiency, recent increases in CO_2_ have already stimulated plant growth, and projected future increases will continue to do so (e.g., Poorter 1993), with the degree of stimulation being at least potentially temperature dependent (Long 1991). Critics of the potential of CO_2_ as a greenhouse-warming gas have stressed that CO_2_-induced stimulation of plant growth will result in a lush plant environment (Idso and Idso 1994); indeed, much of the literature has focused on agronomically important species (see, e.g., Ainsworth et al. 2002; Kimball 1993). However, CO_2_ does not discriminate between desirable (e.g., wheat, rice, and forest trees) and undesirable (e.g., ragweed, poison ivy) plant species with respect to human systems.

## Objectives

What aspects of plant biology currently affect public health? How have, or will, changing levels of CO_2_ and increasing surface temperature change those aspects? For many health care professionals, the role of plant biology has not been fully elucidated, yet it has a number of self-evident impacts, such as nutrition, and perhaps more subtle interactions, such as the spread of narcotic plant species, that deserve our consideration and attention.

## Discussion

### Aerobiology

One of the most common plant-induced health effects is related to aerobiology. Plant-based respiratory allergies are experienced by approximately 30 million people within the United States (Gergen et al. 1987). Symptoms include sneezing, inflammation of nasal and conjunctival membranes, and wheezing. Complicating factors, including nasal polyps or secondary infections of the ears, nose, and throat, may also occur. Severe complications include asthma, cardiac distress, chronic obstructive pulmonary disease, and anaphylaxis.

Quantity and seasonality of pollen are likely to be affected by both climate forcing of phenology and direct effects on pollen production. Overall, three distinct plant-based inputs relate to pollen production: trees in the spring, grasses in the summer, and ragweed (*Ambrosia* spp.) in the fall. In Europe, a 35-year record for birch (*Betula* spp.), a known source of allergenic tree pollen, indicates earlier spring floral initiation and pollen release with anthropogenic warming (Emberlin et al. 2002). At present, the role of seasonality and/or rising CO_2_ on pollen production in grasses remains unknown. Warming has been shown to increase pollen production of western ragweed by 84% (Wan et al. 2002). Initial indoor studies examining the response of ragweed to recent and projected changes in CO_2_ demonstrated an increase in both ragweed growth and pollen production (Rogers et al. 2006; Wayne et al. 2002; Ziska and Caulfield 2000); increased CO_2_ stimulates ragweed pollen production several times more than it stimulates overall growth, and the pollen produced may be more allergenic (Singer et al. 2005). Outdoor experiments that exploited an urban–rural transect also showed the sensitivity of ragweed pollen production to CO_2_ *in situ* (Ziska et al. 2003). In addition, recent research on loblolly pine (*Pinus taeda*) at the Duke University Forest Free-Air CO_2_ Enrichment (FACE) site demonstrated that elevated CO_2_ concentrations (200 μmol/mol above ambient) resulted in early pollen production from younger trees and greater seasonal pollen production (LaDeau and Clark 2006). Besides increased pollen exposure, other consequences of increased fossil fuel burning may be synergistic; for example, diesel particles help deliver aeroallergens deep into airways and irritate immune cells, whereas early arrival of spring and late arrival of fall may extend tree and ragweed allergy seasons, respectively (Ziska et al. 2008a).

Alternatively, more subtle interactions regarding plants may be related to indirect effects of CO_2_ on fungal decomposition. For example, increasing CO_2_ concentration resulted in a 4-fold increase in airborne fungal propagules, mostly spores (Klironomos et al. 1997). The link between spore formation, potential changes in allergenicity of the spores, and the mechanism associated with spore release in the context of elevated CO_2_ has not been entirely elucidated; however, direct effects on microbial function and litter decay seem a likely possibility.

These data suggest a distinct role regarding climate forcing and rising CO_2_ (both at the local urban level, and projected globally) on pollen/spore exposure among the general population. Although the epidemiology of allergic rhinitis is complex, depending on both economic and sociologic factors, the current data also indicate a well-defined role of plant biology in the spread of asthma and respiratory disease. Such associations may help explain the quadrupling of asthma in the United States since 1980 (American Academy of Allergy Asthma and Immunology 2000).

### Contact dermatitis

More than 100 different plant species are associated with contact dermatitis, an immune-mediated skin inflammation. Chemical irritants can be present on all plant parts, including leaves, flowers, and roots, or can appear on the plant surface when injury occurs. One well-known chemical is urushiol, a mixture of catechol derivatives. This is the compound that induces contact dermatitis in the poison ivy group (*Toxicodendron*/*Rhus* spp.). Currently, sensitivity to urushiol occurs in about two of every three people, and amounts as small as 1 ng are sufficient to induce a rash. More than 300,000 people yearly in the United States suffer from contact with members of the poison ivy group (e.g., poison ivy, oak, or sumac) (Mohan et al. 2006). The amount and concentration of these chemicals vary with a range of factors, including maturity, weather, soil, and ecotype. Recent research from the Duke FACE facility also indicated that poison ivy growth and urushiol congeners are highly sensitive to rising CO_2_ (Mohan et al. 2006). Overall, these data suggest plausible links among rising CO_2_, plant biology, and increased contact dermatitis. At present, potential interactions with warmer temperatures and longer growing season in relation to biomass and urushiol content are unknown.

### Toxicology

More than 700 plant species are poisonous to humans. Similar to dermatitis, the presence of toxic substances is related to specific plant organs (fruit, leaf, stem), and edible and poisonous parts can exist on the same plant (e.g., rhubarb, *Rheum rhabarbarum*, and potato, *Solanum tuberosa*). Bracken fern (*Pteridium aquilinum*) may represent a toxicologic threat because of production of potential carcinogenic spores or exudates (Trotter 1990). Poison hemlock (*Conium maculatum*), oleander (*Nerium aleander*), and castor bean (*Ricinus communis*) are so poisonous that tiny amounts can be fatal if ingested (e.g., ricin in castor bean has a greater potency than cyanide). Ingestion of plant material continues to be a very common exposure for humans (particularly children) and can account for nearly 100,000 calls to national poison centers annually (Watson et al. 2004). Pediatric patients comprise more than 80% of plant-related exposures. Only a few plants are associated with potentially life-threatening toxicity, and < 20% of plant exposures require medical treatment (Watson et al. 2004). However, the impact of CO_2_ on the concentration or production of such poisons is almost completely unknown. Rising temperature and longer growing season would, *a priori*, increase the presence of such species in the environment, but, here too, little is known regarding the interaction between CO_2_ and toxicology.

### Pharmacology

Plants have been used for healing since the beginning of civilization. Diversity in the production of secondary chemical products remains an important source of existing and new metabolites of pharmacologic interest (Table 1). Even in developed countries, where synthetic drugs have replaced herbal medicines, 25% of all prescriptions dispensed from community pharmacies from 1959 through 1980 contained plant extracts or active principles prepared from higher plants (e.g., codeine; Farnsworth et al. 1985). For developing countries, however, the World Health Organization (WHO) reported that > 3.5 billion people, or more than half of the world’s population, rely on plants as components of their primary health care (WHO 2002).

**Figure 1:**
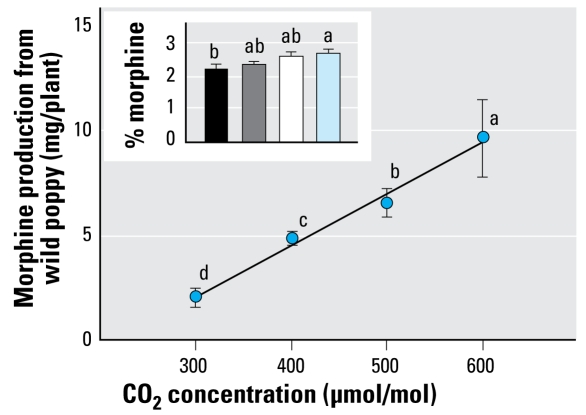
Changes in morphine production and concentration (mean ± SE) from wild poppy (*Papaver setigerum*) as a function of rising levels of atmospheric CO_2_ (Ziska et al. 2008b), corresponding roughly to atmospheric concentrations from 1950, today, and those projected for the years 2050 and 2090, respectively. Different letters indicate significant differences as a function of CO_2_ concentration using Fisher’s protected least significant difference.

Less than 1% of terrestrial plant species have been examined in-depth for their possible pharmacologic use (Pitman and Jorgensen 2002), and only a handful of studies have examined how pharmacologic compounds might respond to recent or projected changes in CO_2_ and/or temperature. Among these, growth of wooly foxglove (*Digitalis lanata*) and production of digoxin were increased at 1,000 μmol/mol CO_2_ relative to ambient conditions (Stuhlfauth and Fock 1990). Production of morphine in wild poppy (*Papaver setigerum*) (Ziska et al. 2008b) (Figure 1) showed significant increases with both recent and projected CO_2_ concentrations. Concurrent increases in growth temperature and CO_2_ also affected the production and concentration of atropine and scopolamine in jimson weed (*Datura stromonium*) (Ziska et al. 2005); however, a synergistic effect on either concentration or production was not observed.

### Food security/nutrition

Adequate diet and nutrition remain key aspects of global health. Among climatic factors, two are likely to have severe consequences for agricultural productivity: water and temperature. Flowering is one of the most thermal-sensitive stages of plant growth (e.g., Boote et al. 2005). Chronic or short-term exposure to higher temperatures during the reproductive stage of development can have negative affects on pollen viability, fertilization, and grain or fruit formation relative to vegetative growth (Hatfield 2008). In addition, water supply, particularly water for irrigation, is at risk with declining ice and snow reserves in mountainous regions (e.g., Kerr 2007). Irrigation is vital to maintaining food security in populous regions in East Asia and elsewhere. Conversely, warmer temperatures and additional CO_2_ could extend growing seasons and boost production; however, there is concern that concurrent increases in CO_2_ and temperature could further exacerbate reproductive sterility because of the indirect effect of CO_2_ on transpirational cooling at the canopy level (Horie et al. 2000; Prasad et al. 2006). With respect to nutrition, plants are anticipated to become more starchy but protein-poor, with a subsequent decline in digestibility as CO_2_ increases (Hesman 2002). In paddy rice, percent protein decreased with both increasing air temperature and higher CO_2_ concentrations over a 2-year period (Ziska et al. 1997). Increasing CO_2_ from preindustrial to current levels resulted in decreased protein in both spring and winter wheat (Rogers et al. 1998); other experiments have also shown a CO_2_-induced reduction in flour protein concentration, as well as changes in optimum mixing time for bread dough, and bread loaf volume (Kimball et al. 2001). Alternatively, strawberries have shown a positive increase in antioxidant capacity and flavanoid content in response to elevated CO_2_ levels (Wang et al. 2003), and mung bean has shown an increase in omega-3 fatty acid content (Ziska et al. 2007).

### Spread of human disease

Plants are not disease vectors per se, but animal reservoirs of disease spread, notably rodents and mosquitoes, rely on plants as a principle food source (although female mosquitoes require blood proteins in order to lay eggs). Given that plant growth, pollen, and seed production among annual plants (including weeds) are likely to increase in response to CO_2_ (Patterson 1995) and warmer temperatures (Wan et al. 2002), greater availability of food supply could result in a higher abundance of these animal vectors, with consequences for disease epidemiology. Pollen on open ponds, for example, can serve as food for mosquito larvae (Ye-Ebiyo et al. 2000); however, it is unclear if CO_2_-induced qualitative changes in pollen (Singer et al. 2005) could also affect mosquito fecundity.

### Pesticide, herbicide, and fungicide use

**Figure 2:**
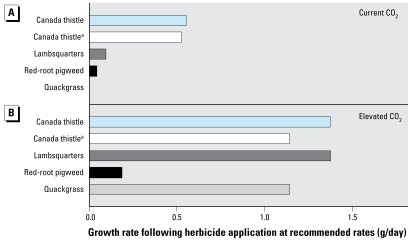
Change in growth rate (g dry matter/day) for weedy species after application of herbicide at recommended doses, when grown at current CO_2_ levels (*A*) and at elevated (600–800 μmol/mol) CO_2_ levels (*B*). At elevated CO_2_ levels (*B*), all growth rates were significantly greater relative to plants that received the same dosage grown at ambient (370–400 μmol/mol) CO_2_ levels (*A*). Herbicide was glyphosate in all cases except where indicated. Increased spraying frequency could overcome CO_2_-induced reductions in efficacy but could increase residual effects within the environment. **^a^**Glufosinate was active ingredient.

Chemical control is the principal means of weed management in most developed countries. Therefore, it is reasonable to ask whether current control efforts could limit any potential or probable impact of climatic forcing or CO_2_-induced changes in plant biology and public health. Temperature and precipitation are known abiotic factors that can affect chemical application rates and overall efficacy (Patterson 1995). There is also evidence from a limited number of studies that rising CO_2_ levels can decrease chemical efficacy for the control of annual and perennial weeds (Figure 2) (Archambault 2007; Ziska and Runion 2007). For Canada thistle, CO_2_-induced reductions in efficacy of glyphosate application were related to greater carbon allocation to roots and a reduction in the systemic effect of the herbicide (Ziska et al. 2004). However, it is not clear if this is a ubiquitous response among perennial weeds. Overall, pests, pathogens, and weeds currently consume some 42% of growing and stored crops annually (Pimentel 1997), and this figure could escalate as a result of higher CO_2_, warming, altered precipitation patterns, and more weather extremes. Increased use of petrochemicals for control carries further risks for human and animal health because it could increase the presence of these chemicals in the environment.

### Uncertainties and limitations

As atmospheric CO_2_ continues to increase, we can expect fundamental changes in plant biology and plant communities, either from anticipated changes in temperature and other abiotic parameters related to climatic forcing, or directly from CO_2_-induced changes in physiology and growth. From the initial studies described here, it is evident that there are a number of plant-based links between such anthropogenic perturbations and public health. Yet, there are a number of key questions that remain to be addressed by the scientific community. What other plant species are likely to increase pollen production in response to CO_2_/temperature increases? How will this affect the epidemiology of allergies/asthma? Will contact dermatitis increase for the general population? Can we expect toxicologic changes in poisonous plants? How will CO_2_-induced changes in food quality affect human nutrition and health? Is the quality or efficacy of plant-based medicines increasing or decreasing? How might CO_2_ and/or climate alter the spread and production of narcotic plants? As plant distribution changes with CO_2_/climate change, how will this affect the ability of mosquitoes or rodents to spread disease? If weed growth is responsive to increasing CO_2_ and increased levels of herbicides are needed for control, how will this affect levels of pesticides in the environment? What steps must we take to ensure food security and adequate nutrition? None of these questions have been addressed in depth; few field data are available that assess both CO_2_ and temperature concurrently with respect to these questions.

## Conclusions

There is a concerted effort among academic and government institutions both to recognize the degree of health risk posed by climate change and to formulate strategies to minimize adverse impacts (for reviews, see Burns 2002; Epstein and Mills 2005; McMichael et al. 2006; Patz and Kovats 2002). However, in these assessments, the role of plant biology in human health has been largely ignored.

We suffer in many ways by what can be called “plant blindness.” That is, when we look at nature, we are more likely to recognize the diversity of animals and only acknowledge plants as a sort of “green background.” Yet, that green background—essential habitat—is highly dynamic. It affects every aspect of our lives, from air, water, clothing to shelter and medicine. The ongoing increase in CO_2_ and its projected impact on temperature and climate represent a clarion call to consider plant interactions beyond the realm of agriculture. Assessing the scale and potential impact of these interactions between plant biology and public health is a facet of human-induced climatic forcing that is underappreciated.

## Figures and Tables

**Table 1:** A partial list of plant-derived pharmaceutical drugs and their clinical uses.

Drug	Action/clinical use	Plant species
Acetyldigoxin	Cardiotonic	*Digitalis lanata* (foxglove)
Allyl isothiocyanate	Rubefaciant	*Brassica nigra* (black mustard)
Artemisinin	Antimalarial	*Artemisia annua* (sweet Annie)
Atropine	Anticholinergic	*Datura stramonium* (jimsonweed)
Berberine	Bacillary dysentery	*Berberis vulgaris* (barberry)
Codeine	Analgesic	*Papaver somniferum* (poppy)
d-Pinitol	Expectorant	Various species
l-Dopa	Anti-Parkinson	*Mucuna pruriens* (velvet bean)
Ephedrine	Antihistamine	*Ephedra sinica* (Mormon tea)
Galanthamine	Cholinesterase inhibitor	*Lycoris squamigera* (surprise lily)
Kawain	Tranquilizer	*Piper methysticum* (kava)
Lapachol	Anticancer, antitumor	*Tabebuia avellandedae* (lapacho tree)
Ouabain	Cardiotonic	*Strophanthus gratus* (climbing oleander)
Quinine	Antimalarial	*Cinchona ledgeriana* (Peruvian bark)
Salicin	Analgesic	*Salix alba* (willow)
Taxol	Antitumor	*Taxus brevifolia* (Pacific yew)
Vasicine	Cerebral stimulant	*Vinca minor* (periwinkle)
Vincristine	Antileukemic agent	*Catharanthus roseus* (Madagascar periwinkle)
